# Church and Sanitation

**Published:** 1897-09-25

**Authors:** 


### CHURCH AND SANITATION.

The Rev. F. Lawrence, speaking at the Sanitary Congress held in Leeds last week, urged that the Church should concern herself with sanitary matters. He pointed out the extent of the Church organisation, how it went into every parish in the land, and what a power for good it might be made if not only the clergy, but all the Church officials were but to unite in an attempt to spread knowledge among the people on sanitary matters. There were, he said, close upon a million Church officials who might become members of the Church Sanitary Association, and might sooner or later be drawn to take a practical part in its work. United effort of the kind foreshadowed, for the advancement of the sanitary creed, may be a long way off, but no one can doubt the enormous effect which such an effort might have in popularising knowledge, and in persuading people of the importance of at least trying to keep in good health.

